# TüEyeQ, a rich IQ test performance data set with eye movement, educational and socio-demographic information

**DOI:** 10.1038/s41597-021-00938-3

**Published:** 2021-06-16

**Authors:** Enkelejda Kasneci, Gjergji Kasneci, Tobias Appel, Johannes Haug, Franz Wortha, Maike Tibus, Ulrich Trautwein, Peter Gerjets

**Affiliations:** 1 Human-Computer Interaction, Department of Computer Science, University of Tübingen, Tübingen, Germany; 2 Data Science and Analytics, Department of Computer Science, University of Tübingen, Tübingen, Germany; 3 Hector Research Institute of Education Sciences and Psychology, University of Tübingen, Tübingen, Germany; 4 Leibniz-Institut für Wissensmedien, Tübingen, Germany

**Keywords:** Education, Education

## Abstract

We present the TüEyeQ data set - to the best of our knowledge - the most comprehensive data set generated on a culture fair intelligence test (CFT 20-R), i.e., an IQ Test, consisting of 56 single tasks, taken by 315 individuals aged between 18 and 30 years. In addition to socio-demographic and educational information, the data set also includes the eye movements of the individuals while taking the IQ test. Along with distributional information we also highlight the potential for predictive analysis on the TüEyeQ data set and report the most important covariates for predicting the performance of a participant on a given task along with their influence on the prediction.

## Abstract (summary)

**Table:** 

Measurement(s)	intelligence • eye movement • Socioeconomic Factors
Technology Type(s)	culture fair intelligence test (CFT-R) • eye tracking device • Questionnaire
Sample Characteristic - Organism	Homo sapiens

Machine-accessible metadata file describing the reported data: 10.6084/m9.figshare.14173244

## Background & Summary

For many decades, research in various fields has been devoted to the question of what constitutes human intelligence^1,2^, the ways in which it develops in the course of our life^3^, and how it can be positively influenced^4^. Additionally, recent developments in the field of artificial intelligence are pushing methodological approaches towards the mimicking of human reasoning and problem solving strategies.

**Fig. 1:**
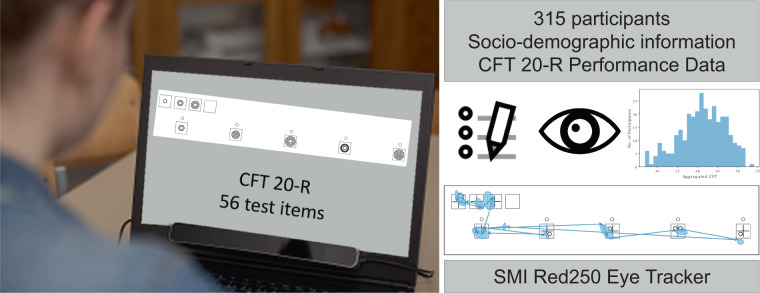
Overview of the TüEyeQ Data Set.

To support the research community and the work at the intersection of psychological and educational sciences and artificial intelligence, we provide the TüEyeQ data set (see Fig. 1). We collected a comprehensive data set from 315 university students performing a culture fair intelligence test (CFT 20-R). The CFT 20-R consists of 4 blocks, each with a fixed time limit and items that successively increase in difficulty. Along with the performance data, we provide socio-demographic and educational background information on the students as well as carefully annotated eye movement data of all participants during task solving. We believe that this data set will boost the research in various fields and will contribute to highly interesting research questions:

- For psychology and psychometric research the question of how perception through eye movements and IQ (as measured by the CFT 20-R test) relate to each other can be thoroughly analysed (e.g.^5^). Also the question which tasks and how many of those tasks are needed to derive the respective conclusions can be explored.
- For educational research, the relationship between an individual’s performance on a standardized IQ Test and socio-demographic and educational background can be further explored.
- From a socio-economic perspective the question of whether the IQ-related performance of a designated and coherent group of people can be boosted by adequately assigning tasks and aggregating answers can be further analyzed (e.g.^6,7^).
- From a data science perspective the data set provides valuable means to analyse performance bias with respect to background information on the participants, such as education, training, viewing behaviour, gender and many more.
- From the cognitive science perspective, this data set can make an important contribution to the study of strategy-related indicators in the context of (complex) problem solving (e.g.^8^). Additionally, TüEyeQ can support research on the relationship between eye movements and (fluid) intelligence (e.g.^9,10^).
- In the research field of human-computer interaction our data set can be used to explore various aspects of cognitive load and adaptation. More specifically, the user performance and behavioral data as described by the eye movements and pupillary measures can be employed to identify indicators of cognitive load and how they can be used to predict task difficulty under time constraints.
- From the AI perspective the question of whether machine learning algorithms can learn to reason as humans and whether it is possible to develop an AI system that correctly solves such a test is among current challenges in AI research (e.g.^11–13^).

## Methods

### Experimental design description

For TüEyeQ, 315 healthy participants (217, female, 94 male, 4 not stated; with an age mean of 23.272 years, SD 3.022) with a university entrance qualification, without neurological or psychiatric pre-existing conditions, and no visual impairment above 3 dioptres participated in our study. All participants underwent a large-scale study which aims at investigating the interaction between different partial abilities relevant for self-regulation in educational contexts. For this purpose, 321 participants were recruited. They performed different cognitive tasks (measuring, for instance, executive functioning or IQ) and filled in multiple questionnaires regarding self-regulation indicators (e.g., motivational beliefs or personality traits) in a lab setting during three sessions, each lasting up to 4 hours. Unfortunately, we had to exclude 4 participants because they did not complete all study sessions, and two more participants were excluded because of technical issues. For their participation in the study, the participants received remuneration of 8 EUR per hour and additionally 15 EUR in case they participated in all sessions. The participation in the study could be revoked at any time. All collected data were anonymized and treated confidentially.

In this work, we report specifically on performance data of this cohort of participants in an IQ test, their eye movement behavior during solving this IQ test and their socio-demographic and educational background characteristics, including information on software usage and leisure time activities. The questionnaire on socio-demographic and educational background of the participants was the first questionnaire of the first session of the study. The participants completed the CFT test immediately afterwards.

The Ethical Review Board of the Psychological Institute at the University of Tübingen approved the protocol of the study. All participants were informed in written form and consented that their anonymous data can be analyzed and published. Due to a self-constructed pseudonym, they had the option to revoke this consent at any time.

### The CFT 20-R test

**Fig. 2:**
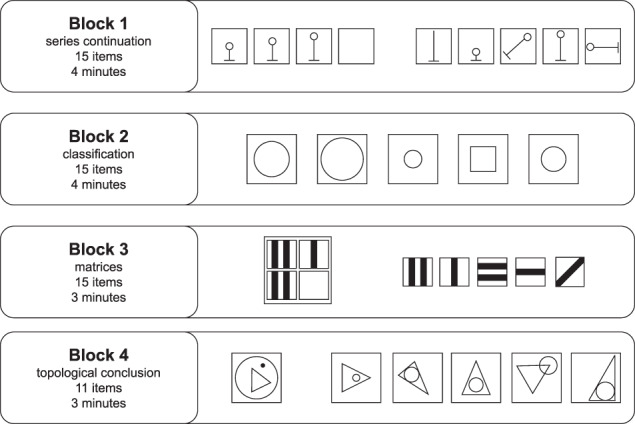
Example test items from the CFT 20-R test as employed in our experiment.

In our experiment, we employed the first part of the revised version of the culture fair intelligence test (CFT-R) designed by Weiß *et al*.^14^. This IQ-test is intended to measure the general mental capacity (i.e., the g-factor of intelligence or fluid intelligence) by means of tasks that require the ability to recognize figural relationships and to engage in formal logical thinking in problems of varying degrees of complexity under time restriction. Since the tasks administered are language-free, it is assumed that people with poor language knowledge and poor cultural techniques are not disadvantaged. The CFT 20-R consists of four blocks of different tasks, namely series continuation, classifications, matrices and topological conclusions. Each of these blocks has 11–15 items with increasing difficulty and a time limit of 3–4 minutes. Details and exemplary test items are presented in Fig. 2.

In order to record the eye movements of the participants during the task, we adapted the classic pen-and-paper version of the IQ test to a digital one that can be displayed on a computer screen. To be as close to the paper version as possible, we presented as many items as possible on a single screen page as long as this did not necessitate scrolling.

Participants first received general instructions about the nature of the test, followed by the first block. Each block had a specific instruction, introducing the participants to the requirements of this block and demonstrating the essence of the task based on 3 examples. The instruction phase was conducted without time constraints, thus all participants could go through the examples and make themselves familiar with the test procedure. All instructions were presented in German using the SoSci Survey online platform.

### Data acquisition

Data collection took place in a digital classroom equipped with 30 remote eye trackers attached to laptops with 17inch HD display screens running at full brightness with a resolution of 1920 × 1080. This setup allows for data collection of up to 30 participants simultaneously, minimizing the overall time needed for collection. For this study, verbal instructions were given en masse pertaining to a brief overview of the protocol and an explanation of eye tracking, then individual calibrations were performed with a supervised quality check. Interactions between the participants and the computer took place via mouse or touch pad, depending on participants’ preference. The distance between participants and their respective screen was 50–70 cm depending on participants’ preferences.

The collection environment had the room illumination level controlled with no effects from sunlight or other outdoor light. The standard maintained illuminance for the experimental sessions was between 10 to 50 lux, measured with a Lux sensor (i.e., Gossen Mavo-Max illuminance sensor, MC Technologies, Hannover, Germany).

Eye movement data was collected by means of SMI RED250 remote eye trackers, a commercial eye tracker with 250 Hz sampling frequency. Since the eye tracker has a high sampling frequency, both stable (fixations) and rapid (saccadic) eye movements for static stimuli can be measured. Eye movements were recorded using the included eye-tracking software Experiment Center which outputs the raw gaze data consisting of x and y coordinates of each data point, the timestamp information and the pupil diameter in millimeters.

Calibration was performed for all participants using SMI’s built in 9-point calibration. A validation also was performed as a quality check to measure the gaze deviation for both eyes from a calibration point: A deviation larger than one degree required re-calibration. Calibrations were performed prior to the experiments as well as one or two times during the experimental session, depending on how many images were presented.

## Data Preprocessing

### Quality of eye tracking data

Initially, the raw gaze data was examined for signal quality using the eye-tracking software BeGaze provided along with the eye trackers. This software reports proportion of valid gaze signal to stimulus time as the tracking ratio. Therefore, if a participant’s tracking ratio was deemed insufficient (i.e., less than 80% for at least a part of the task), we omitted his or her data. This pre-processing stage can assure that errors (e.g. post-calibration shifts, poor signal due to glasses) in the gaze data are substantially minimized. Consequently, eye-tracking data of 58 participants had to be omitted due to low tracking ratios. Further 11 data sets were excluded due to errors in the presentation software and another 17 because of incomplete data. This leaves us with eye tracking data for only 229 of 315 participants. The raw eye-tracking data was then pre-processed to improve the data quality and to extract several features.

### Fixations

Fixations are periods where the eye is stationary and a single location is gazed upon usually lasting between 200 ms and 350 ms^15^. Fixations not only contain information regarding what exact location participants look at, but also provide useful characteristics like frequency and duration. Longer fixations are associated with higher processing load and more effort^16,17^. As a further consequence, frequency of fixations increases as their duration increases. Fixation information was extracted from the eye-tracking protocols based on the I-VT algorithm^18^ as implemented by the Perception’s Engineer Toolkit^19^. As a minimum fixation duration a threshold of 75 *ms* was chosen.

### Saccades

Saccades are rapid eye movements that allow to change the focus of attention. As for fixations, saccades were extracted from the eye-tracking protocols based on the I-VT algorithm^18^ with a velocity threshold of 30°/s. Since the velocity of saccades is not voluntarily controlled^20^, but depends on the neural activity, our data provides a powerful resource to study gaze parameters in cognitive engagement.

### Microsaccades

Microsaccades are fixational eye movements, which occur during an especially prolonged fixation. They are similar to regular saccades, but smaller, jerk-like, and happen involuntarily. Their properties are linked to visual attention^21,22^, perception^23^, working memory^24^, and task difficulty^25^. To identify microsaccades we followed the procedure proposed by Krejtz *et al*.^26^. The reported microsaccade-related features are comprised of the occurrence of microsaccades per fixation, their mean peak velocity, and their mean amplitude.

### Pupil-related features

Pupil diameter has been used as an indicator of cognitive load for a very long time. Already in 1964, Hess and Polt reported that increasing task difficulty lead to increased pupil diameters^27^. Since then, this finding has been replicated across many different domains including short-term memory, language processing, reasoning, perception, sustained attention and selective attention^28–30^. Pupil dilation, however, is not only invoked by cognitive load^31^, but also emotional arousal^32^ or lightning conditions, fatigue^33,34^, pain^35^, and distance to a fixated object^36^. A comprehensive overview of factors effecting the pupil is provided by Mathot^37^.

To enable further research in the above mentioned areas based on our IQ-Data, we pre-processed the recorded pupil signal to enhance its quality and reduce the influence of artifacts and noise. For example, blink artifacts were removed by removing eye-tracking data in a time window 100 ms before and after a blink. During this period, the pupil may still be effected by the blink and recorded diameters are unlikely to accurately reflect any of the above mentioned effects (e.g., cognitive load, fatigue, etc). Furthermore, we removed pupil diameter values smaller than 0 (i.e., measurement errors) and replaced them by missing values. Finally, we interpolated small gaps of up to 50 ms (which corresponds to 12 data points at a sampling rate of 250 Hz) and smoothed the data using a third order low-pass Butterworth filter with a 2 Hz cutoff as proposed by^38^.

From the processed pupil signal, we extract the mean which is provided as an additional feature along with fixation and saccade information.

### Visual scanpath annotation

**Fig. 3:**
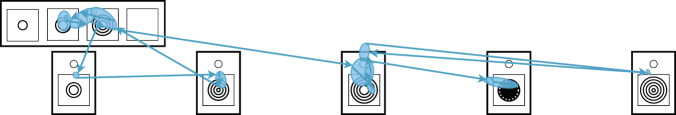
Schematic overview of the annotated areas of interests and the overlaid visual scanpath.

For each test item, we carefully annotated the visual search behaviour as the spatio-temporal sequence of fixations and saccades, i.e. the visual scanpath, during task solving. Figure 3 shows an exemplary test item as included within Block 1 of the CFT-R test. The participant has to find the best matching shape (i.e., target) among four distractors, which provides the best continuation of the given shape series. Semantic annotations in so-called Areas of Interest (AoIs) is visualized with a black box around specific parts of the stimulus. The figure further features an exemplary scanpath of a participant, where fixation locations are visualized by ellipses and saccades as arrows or vectors.

**Fig. 4:**
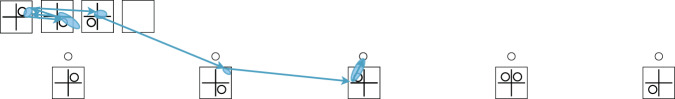
Visual scanpath for a task that was solved correctly and with high certainty.

**Fig. 5:**
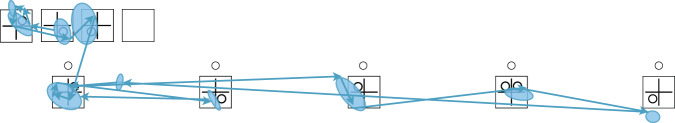
Visual scanpath for the same task as in Fig. 4 for the case of a wrong solution and high uncertainty.

Figures 4 and 5 show the visual scanpaths of two participants solving the same task. The participant, whose visual scanpath is shown in Fig. 4, finds the solution (target) within very few fixations. Since the further options are not even visually explored, we can argue that the task was solved at high certainty. In contrast, the visual scanpath of the second participant, depicted in Fig. 5, reveals a visual exploration strategy which reflects a high uncertainty and anticipates a wrong solution to the given task. This example highlights the potential for further in-depth analysis of visual scanpaths and other eye movement measures related to (fluid) intelligence.

### IQ task performance data

Performance in the CFT was dichotomized for each item, where one corresponds to a correct solution and zero to an incorrect solution. We decided to indicate items where no answer was selected as missing values because these missing values convey additional information (e.g., items that were skipped are identifiable trough this coding). Due to technical problems, six items in the CFT had issues in a distractor or target. Specifically, the eleventh item from the matrices and the third item from the topological conclusions block displayed an erroneous target during the experiment (i.e., the correct answer was not included in the possible options). The four other items (from the six with technical issues) included an inaccurate distractor. To reconcile these issues and maintain the validity of the overall task performance multiple steps were taken. First, all affected items were marked in the data set (suffix ‘e’). Second, the two items with erroneous targets were treated as missing, as participants were not able to solve them correctly. Third, sum scores for the items with inaccurate distractors, the regular items, and both, were compared through correlations. The analyses showed that performance on the items with inaccurate distractor and performance on the correctly displayed items (r(315) = 0.412, p < 0.001) as well as the overall sum scores with and without items with inaccurate distractors (r(315) = 0.992, p < 0.001) correlated significantly. This indicates that the items with an inaccurate distractor still measure performance that corresponds to overall task performance and are eligible for further analyses.

### Socio-demographic Information

In addition to the above mentioned performance and eye movement features, we report socio-demographic and educational background information for each participant. The background information also includes leisure and gaming activities, software and internet usage, programming experience, and many more. A detailed description of these features along with their representation (i.e., encoding) in the data set is provided in Table 1.

**Table 1:** Description and encoding of all performance-related, educational and socio-demographic features in the order of their appearance in the csv file.

Variable Nr.	Feature	Description	Encoding
1	task_ID	Unique identifier for every task	String, CFT-block-related task id
2	participant	Unique identifier for every participant	String-based id
3	age	The age of a participant	categorical
4	gender	The gender of a participant, i.e. male, female, unknown	categorical
5	handedness	Indicates whether the participant is right-handed or left-handed	binary
6	native_german	This variable describes whether a participant is a native German	binary
7	native_german_mother	Indicates whether the mother of the participant is a native German	binary
8	native_language_mother	The native language of the participant’s mother	categorical
9	native_german_father	Indicates whether the father of the participant is a native German	binary
10	native_language_father	The native language participant’s father	categorical
11	education_mother	The scholarly or professional education of the participant’s mother	categorical
12	education_father	The scholarly or professional education of the participant’s father	categorical
13	training_mother	The scholarly or professional training of the participant’s mother	categorical
14	training_father	The scholarly or professional training of the participant’s father	categorical
15	books	Indicates how many books are in the participant’s household	categorical
16	job_mother	The profession of the participant’s mother	categorical
17	job_father	The profession of the participant’s father	categorical
18	year_of_degree	The year in which the final study degree was achieved by the participant	categorical
19	mean_grade_degree	The average grade of the participant’s final degree	continuous
20	programming_experience	Indicates whether the participant has experience programming languages	binary
21	smartphone_usage	Indicates the frequency of smartphone usage (range: never to daily)	categorical
22	tablet_usage	Indicates the frequency of tablet usage (range: never to daily)	categorical
23	notebook_usage	Indicates the frequency of notebook usage (range: never to daily)	categorical
24	desktop_pc_usage	Indicates the frequency of desktop pc usage (range: never to daily)	categorical
25	tv_usage	Indicates the frequency of tv usage (range: never to daily)	categorical
26	text_editor_usage	Indicates the frequency of text editors usage (range: never to daily)	categorical
27	spreadsheet_usage	Indicates the frequency of spreadsheet software usage (range: never to daily)	categorical
28	presentation_software_usage	Indicates the frequency of presentation software usage (range: never to daily)	categorical
29	email_usage	Indicates the frequency of email usage (range: never to daily)	categorical
30	browser_usage	Indicates the frequency of web browser usage (range: never to daily)	categorical
31	google_usage	Indicates the frequency of Google usage (range: never to daily)	categorical
32	wikipedia_usage	Indicates the frequency of Wikipedia usage (range: never to daily)	categorical
33	facebook_usage	Indicates the frequency of Facebook usage (range: never to daily)	categorical
34	twitter_usage	Indicates the frequency of Twitter usage (range: never to daily)	categorical
35	skype_usage	Indicates the frequency of Skype usage (range: never to daily)	categorical
36	youtube_usage	Indicates the frequency of Youtube usage (range: never to daily)	categorical
37	ebay_usage	Indicates the frequency of Eabay usage (range: never to daily)	categorical
38	amazon_usage	Indicates the frequency of Amazon usage (range: never to daily)	categorical
39	online_news_usage	Indicates the frequency of online news usage (range: never to daily)	categorical
40	online_banking_usage	Indicates the frequency of online banking usage (range: never to daily)	categorical
41	gaming_adventure	Indicates whether the participant primarily plays adventure games	binary
42	gaming_action	Indicates whether the participant primarily plays action games	binary
43	gaming_first_person_shooter	Indicates whether the participant primarily plays first person shooter games	binary
44	gaming_casual	Indicates whether the participant primarily plays casual games	binary
45	gaming_mmo	Indicates whether the participant primarily plays Massive Multiplayer Online games	binary
46	gaming_racing	Indicates whether the participant primarily plays racing games	binary
47	gaming_rpg	Indicates whether the participant primarily plays Role Playing Games games	binary
48	gaming_simulation	Indicates whether the participant primarily plays simulation games	binary
49	gaming_sports	Indicates whether the participant primarily plays sports games	binary
50	gaming_strategy	Indicates whether the participant primarily plays strategy games	binary
51	smoking	Indicates whether the participant is a smoker	binary
52	excessive_drinking	Indicates whether the participant is an excessive drinker	binary
53	grades_math	The participant’s final math grade (German Abitur)	continuous
54	grades_german	The participant’s final German grade (German Abitur)	continuous
55	grades_biology	The participant’s final biology grade (German Abitur)	continuous
56	grades_physics	The participant’s final physics grade (German Abitur)	continuous
57	grades_chemistry	The participant’s final chemistry grade (German Abitur)	continuous
58	grades_geography	The participant’s final geography grade (German Abitur)	continuous
59	grades_history	The participant’s final history grade (German Abitur)	continuous
60	grades_art	The participant’s final art grade (German Abitur)	continuous
61	gaming_hours_weekly_min	The minimum hours the participant spends gaming per week	continuous
62	gaming_hours_weekly_max	The maximum hours the participant spends gaming per week	continuous
63	leisure_simple_entertainment	Indicates whether the participant’s leisure activity involves simple entertainment	binary
64	leisure_mental_activity	Indicates whether the participant’s leisure activity involves mental activity	binary
65	leisure_sports_exercise	Indicates whether the participant’s leisure activity involves sports and exercise	binary
66	leisure_music	Indicates whether the participant’s leisure activity involves music	binary
67	leisure_art	Indicates whether the participant’s leisure activity involves art	binary
68	leisure_dance	Indicates whether the participant’s leisure activity involves dance	binary
69	leisure_hobbies	Indicates whether the participant’s leisure activity involves hobbies (e.g. DIY)	binary
70	leisure_play_games	Indicates whether the participant’s leisure activity involves playing (video-) games	binary
71	leisure_relaxation	Indicates whether the participant’s leisure activity involves relaxation	binary
72	leisure_social_activity	Indicates whether the participant’s leisure activity involves social activities	binary
73	leisure_humanitarian_services	Indicates whether the participant’s leisure activity involves humanitarian work	binary
74	leisure_nature_activities	Indicates whether the participant’s leisure activity involves nature/outdoor activities	binary
75	leisure_travel_tourism	Indicates whether the participant’s leisure activity involves travel and tourism	binary
76	study_subject_primary	The primary study subject category of the participant	categorical
77	study_subject_secondary	The secondary study subject category of the participant	categorical
78	cft_sum_full	The aggregated CFT score of the participant	continuous
79	cft_task	Indicates whether the participant solved the task correctly	binary

To enable comparisons with related studies, we categorized the string variables “job_father”, “job_mother”, “leisure”, and “study_subjects” according to common taxonomies.

Specifically, we have categorized the leisure activities according to^39^, who distinguish between 13 recreational activities. Since most of our participants take part in several leisure activities, we mapped the activities to a one-hot encoding scheme, which corresponds to all features with the prefix “leisure_[…]” in the data set.

The International Standard Classification of Education (ISCED) provides a framework of definitions that enable a comparison of education systems. We categorized our “study_subjects” according to the Fields of Education and Training^40^ of ISCED.

Similarly, the International Standard Classification of Occupations (ISCO-08)^41^ defines different groups of occupations. However, we found that ISCO-08 does not adequately represent the range of occupations that was provided by our German participants. Specifically, ISCO-08 would have introduced a significant imbalance with respect to the “Professionals” category. Instead, we applied the taxonomy of stepstone.de (as of Nov. ’20), which is one of the most popular online job markets in Germany. This taxonomy led to a much more fine-grained and up-to-date categorization in “job_father” and “job_mother”.

## Data Records

The TüEyeQ data set is available through the Harvard Dataverse Repository^42^.

The performance data along with the socio-demographic and education background information is provided as a single csv-file (cft-full) with 17,010 rows (observations) and 79 columns (features). For each of these features, Table 1 provides both the corresponding description and the encoding.

Eye movement information is provided in the folder (EyeMovementData) and comes in three formats. The first is a participant- and stimulus-wise raw format in folder (raw) that has only been pre-processed as described in the Section Data Preprocessing and consequently contains features for each fixation and saccade. Each subfolder here contains the raw eye-movement data for the participant with the participant ID being the same as the subfolder name. The therein contained files are named according to the corresponding stimulus (e.g., 1–3.csv means that this screen page contains the tasks 1 to 3 from the CFT test). Furthermore, a schematic version of each page of the CFT is provided in the folder (stimuli). For copyright reasons, this folder contains only the AOIs, the specific content of which can be retrieved from the CFT 20-R test^14^.

Mouse clicks of all participants are available in (clicks) – a csv file offering data about each click that was performed by a participant, describing thus, when and where a mouse click occurred, as well as on which part of what IQ task. This information is important for data analysis, since it details when a participant chose an answer and when he or she changed it.

In addition to the raw gaze data, we provide a convenience format stored in the folder (split), where the eye movement data is split up task-wise. This format features folders with participants’ IDs that hold task-wise data (e.g., task_07.csv for the seventh task of the CFT 20-R). For this convenience format, participants with substantial offsets in their fixations or noise that could indicate extensive head movements were removed after a visual inspection of the raw eye movement data. This accounted for the removal of 43 participants reducing the set to 186. Most of these 43 participants may be included after manual correction of the fixations at the reader’s discretion. The procedure for the assignment of events to tasks is described in short as follows:

- Areas deemed relevant were the areas of each sub-task as well as the time remaining and the overall progress bar.
- Any other area was considered task-irrelevant
- Fixations on task-irrelevant AoIs were removed
- Saccades that neither started nor ended on a relevant AoI were removed
- Event outliers were removed by considering the 5 events before and after this event. If less than 30% if these events shared the same AoI, the fixation was considered an outlier.
- All events on the AoIs of a task that happened after the participant had performed their final click for that task were removed.
- Events involving either the progress bar or the timer were assigned to the task that had the most recent valid gaze.
  For each participant, the eye movement information is provided as cvs files which contain the eye movement and pupil features. All coordinates have their origin in the top left corner of the screen and the x and y axis extend to the right and bottom respectively. The csv files contain the following features:
- starttime: Timestamp information
- duration: Duration of the event
- meanPupilDiameter: Only for fixations, mean pupil diameter
- eye: Left or right eye
- eventType: Fixation or saccade
- eventIdxLeft: Index of left eye event
- eventIdxRight: Index of right eye event
- meanX: Only for fixations, mean X coordinates
- meanY: Only for fixations, mean Y coordinates
- startSaccadeX: Only for saccades, X coordinates of saccade starting point
- startSaccadeY: Only for saccades, Y coordinates of saccade starting point
- endSaccadeX: Only for saccades, X coordinates of saccade ending point
- endSaccadeY: Only for saccades, X coordinates of saccade ending point
- microsaccadeCount: Only for fixations, number of microsaccades
- microsaccadeAmplitide: Only for fixations, mean amplitude of microsaccades
- microsaccadePeakVelocity: Only for fixations, mean peak velocity of microsaccades

Eye movements and mouse clicks share the same timestamp format, which makes them easy to synchronize for analysis. Furthermore, eye movements, AoIs, and mouse clicks share a common coordinate system allowing for convenient use of all three data sources.

Finally, we provide aggregated eye-tracking data for each participant and task that is already included in the ready-to-use format. This data is contained in the file named eye_features_aggregated.csv and consists of mean values for each participant and task. For each participant and task, this file provides mean values of fixation duration, saccade count, saccade amplitude, saccade duration, microssacade count, microsaccade amplitude, microsaccade duration, microsaccade peak velocity, and pupil diameter.

## Technical Validation

### Bias considerations

**Fig. 6:**
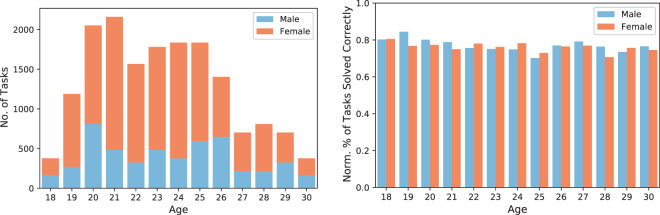
Histograms on the age distribution over all tasks (on the left), and normalized by all correctly solved tasks (on the right). We grouped both histograms according to the gender of the corresponding participants (represented by the colors).

As Fig. 6 shows, there is neither an age-related nor a gender-related bias with respect to the overall performance over all tasks. More specifically, there is no significant difference in performance between male and female participants across the different age groups (right plot in Fig. 6). The four participants whose gender was unknown were omitted in this analysis.

### Distribution of the aggregated CFT score

**Fig. 7:**
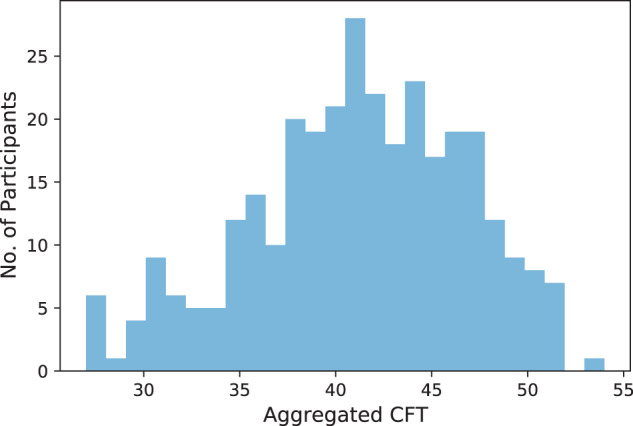
Histogram of the aggregated CFT scores (i.e., *cft_sum_full*). We separated the scores into 26 bins, since this is the number of unique aggregated CFT scores in the data.

Also, as shown in Fig. 7, the overall performance of the participants – encoded by the variable *cft_sum_full* – is, as expected, approximately normally distributed. As indicated by the distribution, there are 7 participants (with participant IDs: ‘AAB14’, ‘ACB13’, ‘ATT30’, ‘BUO15’, ‘OAK22’, ‘SKA02’, ‘VWK01’) – represented by the two bars on the very left-hand side of the distribution plot - who show an unexpectedly low overall performance. We hypothesize that these participants did not take the experiment seriously or simply went through the tasks without thinking carefully about the possible solutions. Many of the CFT tasks were left unsolved by these participants.

### Performance distribution over the CFT tasks in the order of their appearance

**Fig. 8:**
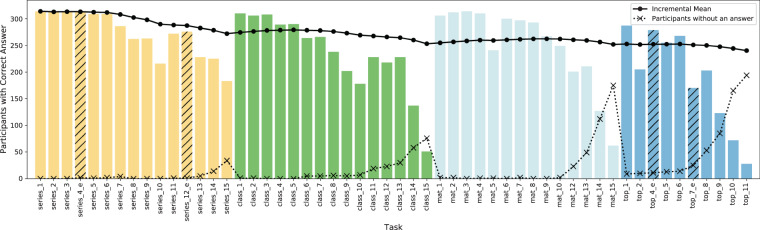
Histogram of correctly solved tasks. The dotted line with the x-markers show the number of missing answers per task (the dots have only been added for greater clarity). The continuous line is the incremental expected number of participants who can solve any given task from the ones seen so far correctly. For example, at the final task, *top_11*, we expect 240 out of 315 participants to solve a given task correctly. More specifically, after observing all tasks, each task has been solved correctly by 76.2% of the participants on average. The four task tasks with negligible technical issues during the data collection are highlighted by the four diagonally crosshatched bars.

Figure 8 shows a histogram over all solved tasks in the order of their appearance in the CFT 20-R. Moreover, for every task, we show the incremental expected number of participants who can correctly solve a given task from the ones seen so far. We see that while the incremental mean decreases, the number of tasks that were skipped by the participants increases, especially within CFT blocks. This is in accordance with the intuition that CFT tasks are ordered (especially within the CFT blocks) by increasing degree of difficulty.

### Discriminative information for predicting a participant’s performance on a given CFT task

In order to show that our data set does indeed contain discriminative information with respect to a participant’s performance on a given CFT task, we performed a binary classification on *cft_task* using the socio-demographic features. To this end, we ignored all observations with missing values (1,248 entries), i.e. all tasks, where no answer was provided. In general, however, these missing values carry semantic meaning and may be worth further investigating. Besides, we removed the feature *participant* because it represents an identifier and as such it inadequately increases the dimensionality of the feature vectors. The variable *task_id*, however, despite representing an identifier, reflects the order in which the CFT tasks occur and thus encodes the difficulty of tasks. Since CFT tasks are ordered by increasing order of difficulty within each CFT block and across blocks, we expect *task_id* to be a highly discriminative feature with respect to the performance of a participant on a given CFT task. We also removed *cft_sum_full*, which is an aggregation of the target and could thus cause information leakage.

We applied the following pre-processing steps:

- We factorized the string features (i.e. we mapped them to integers).
- We imputed the NaN-values in all categorical features with a new category.
- We imputed the NaN-values in all continuous features with their median.
- Additionally, we normalized all continuous features to the interval [0,1], by using the MinMaxScaler of scikit-learn. The normalization was required for the Logistic Regression experiment, since we applied an $\ell$2 regularization.

We randomly split the data into a training (80%) and test set (20%) and trained a Logistic Regression model on TüEyeQ. We specified an $\ell$2 regularization and a maximum of 1,000 iterations to train the Logistic Regression model.

**Fig. 9:**
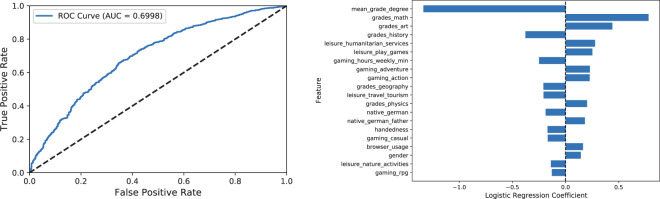
The Receiver Operating Characteristic curve (left) and the highest coefficients (right) of a Logistic Regression model for predicting a participant’s performance on a given task. More specifically, the model is aimed at binary classification of “correct” and “incorrect” answers (where *cft_task* was used as the target variable).

**Fig. 10:**
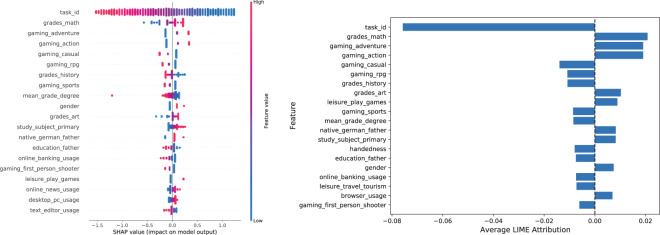
Shapley values corresponding to a Logistic Regression model (left) and Lime explanations (right). The Shapley and Lime values correspond to the ROC curves in Fig. 9. We used LinearSHAP and LimeTabular for the Logistic Regression.

To illustrate the importance of input features for the prediction (see Figs. 9 and 10), we computed Shapley values according to^43^ and Lime values according to TabularLime^44^. Specifically, we approximated Shapley values with LinearSHAP^43^ for the Logistic Regression model. Furthermore, we also report the coefficients of the Logistic Regression model. Figure 10 and 9 (right) show that there tends to be an agreement between Shapely values, the Lime attributions and the Logistic Regression coefficients with respect to the variable influences on the scores produced by the Logistic Regression model. More specifically, variables like *task_id* (which implicitly encodes the task difficulty) and *grades_math* along with other grades and variables related to gaming, online and leisure activities are assigned by all approaches high absolute scores, indicating a high importance of these aspects for the prediction model. Especially the importance of the gaming-related variables in the model is in alignment with recent research investigating the relationship between gaming and fluid intelligence (e.g.^45^), thus providing through TüEyeQ new means for further analysis of such relationships in a thorough way including eye movement behavior.

### Variables with highest correlation to the aggregated CFT score

Interestingly, as shown in Table 2, the two variables with the strongest Distance Correlation^46^ to the aggregated CFT score, *cft_sum_full*, are *grades_math* and *mean_grade_degree*. This is in alignment with the importance attribution results discussed above, where these two variables were assigned a high importance for the Logistic Regression model. However, in addition, we also see that other variables like *grades_chemistry* or *grades_physics*, which were not regarded as important by the previous attribution schemes, are among the variables with the highest correlation to *cft_sum_full*. These results together with those discussed in the previous paragraph show that the analysis of the variables’ impact on the performance in an IQ test can be quite intricate and needs to involve different measures and cover various aspects.

**Table 2:** Variables with the highest Distance Correlation^46^ to the aggregated CFT score (*cft_sum_full*).

Feature 1	Correlation with *cft_sum_full*
grades_math	0.34335
mean_grade_degree	0.29253
grades_chemistry	0.24498
grades_physics	0.22522
native_german_father	0.21195
education_father	0.20961
cft_task	0.20898
native_language_father	0.20001
grades_biology	0.18949
programming_experience	0.17870
spreadsheet_usage	0.14477
native_german_mother	0.13621
native_german	0.13383
text_editor_usage	0.13258
education_mother	0.13026
leisure_travel_tourism	0.12710
grades_art	0.12149
age	0.12012
grades_german	0.11945
training_father	0.11916

### Correlation between variables

**Fig. 11:**
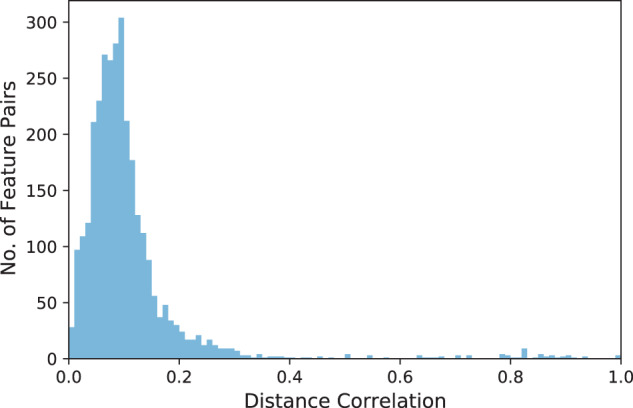
Histogram showing the Distance Correlation^46^ between features. We grouped the correlation scores into 100 bins.

The correlation scores in Table 3 correspond to the pairwise Distance Correlation coefficient for a random sample of 20% of the observations. Note that the Distance Correlation measures both linear and non-linear relationships between two random vectors. As depicted in Fig. 11, the pairwise correlations are centered around 0.1. The peaked distribution indicates that most of the variable pairs are uncorrelated or very weakly correlated. As shown in Table 3, only 54 out of 3,081 variable pairs have a Distance Correlation above 0.6 (and only 62 pairs have a correlation above 0.5). For the Logistic Regression model described above, we did not remove highly correlated variables, which might entail collinearity issues. Hence, we believe that better predictability could be achieved if more effort was invested in the data analysis and preprocessing steps.

**Table 3:** All pairs of variables with a Distance Correlation^46^ above 0.6.

Feature 1	Feature 2	Correlation
gaming_mmo	gaming_racing	1.00000
smoking	excessive_drinking	1.00000
gaming_hours_weekly_min	gaming_hours_weekly_max	0.99517
gaming_adventure	gaming_action	0.93618
gaming_action	gaming_casual	0.93020
gaming_casual	gaming_rpg	0.91362
gaming_action	gaming_sports	0.90698
gaming_adventure	gaming_sports	0.90695
gaming_casual	gaming_sports	0.90007
gaming_action	gaming_rpg	0.89854
gaming_adventure	gaming_casual	0.89358
gaming_casual	gaming_strategy	0.88825
gaming_adventure	gaming_rpg	0.87973
gaming_rpg	gaming_sports	0.87833
gaming_action	gaming_strategy	0.87555
gaming_rpg	gaming_strategy	0.86418
gaming_sports	gaming_strategy	0.86186
native_german_father	native_language_father	0.85660
gaming_casual	gaming_racing	0.85643
gaming_casual	gaming_mmo	0.85643
gaming_adventure	gaming_strategy	0.85103
gaming_casual	gaming_simulation	0.84677
gaming_action	gaming_mmo	0.82699
gaming_action	gaming_racing	0.82699
gaming_racing	gaming_rpg	0.82624
gaming_mmo	gaming_rpg	0.82624
gaming_adventure	gaming_racing	0.82463
gaming_adventure	gaming_mmo	0.82463
gaming_racing	gaming_sports	0.82424
gaming_mmo	gaming_sports	0.82424
gaming_action	gaming_simulation	0.82163
gaming_rpg	gaming_simulation	0.81015
gaming_adventure	gaming_simulation	0.80030
gaming_simulation	gaming_sports	0.79692
native_german_mother	native_language_mother	0.79633
gaming_simulation	gaming_strategy	0.79156
gaming_racing	gaming_strategy	0.78592
gaming_mmo	gaming_strategy	0.78592
gaming_racing	gaming_simulation	0.78168
gaming_mmo	gaming_simulation	0.78168
native_german_mother	native_german_father	0.72413
gaming_first_person_shooter	gaming_rpg	0.72325
mean_grade_degree	grades_math	0.72119
gaming_adventure	gaming_first_person_shooter	0.70774
gaming_action	gaming_first_person_shooter	0.70380
gaming_first_person_shooter	gaming_casual	0.70012
native_german_mother	native_language_father	0.67905
gaming_first_person_shooter	gaming_sports	0.67386
native_language_mother	native_language_father	0.66508
gaming_first_person_shooter	gaming_strategy	0.65446
gaming_first_person_shooter	gaming_simulation	0.64308
gaming_first_person_shooter	gaming_racing	0.63502
gaming_first_person_shooter	gaming_mmo	0.63502
native_language_mother	native_german_father	0.63094

## Usage Notes

The data can be downloaded from 10.7910/DVN/JGOCKI as a csv file. All experiments are provided as a Jupyter notebook and may therefore be easily reproduced.

When using the TüEyeQ dataset or parts of it, please cite this manuscript.

Besides the data provided in this work, the authors can provide access to further variables and performance data of the participants on other tasks upon request. Such data comprises for example information on the “Big Five” personality factors, on internal and external locus of control, self-efficacy, attribution styles, academic self-concepts, domain-specific interests, motivational strategies, process and questionnaire data on learning success, learning processes and learning prerequisites as well as performance data for test items measuring executive control functions such as, flexibility, updating, inhibition (n-back, operation and reading Span items, Stroop item performace, Stop Signal Test, Trail Making Test). In this case, a sub-selection of the dataset, whose size will be defined based on the specific requests from interested users and on the processing time needed will be provided. For any questions, suggestions or request of collaboration regarding TüEyeQ please contact the corresponding author. This data set is freely available under the CC0 license.

## Data Availability

All Python-code corresponding to the evaluations described in this work is distributed on GitHub under the MIT license https://github.com/haugjo/TueEyeQ. To run the evaluation, the following packages are required (note that older or more recent versions might also work): • python (v3.7.3) • numpy (v1.18.1) • pandas (v0.25.1) • scikit-learn (v0.21.3) • matplotlib (v3.1.3) • shap (v0.34.0) • lime (v0.2.0.1) • dcor (v0.5.2) • pickleshare (v0.7.5, only required to load the precomputed Distance Correlation scores).
